# Aberrant pre-mRNA processing in cancer

**DOI:** 10.1084/jem.20230891

**Published:** 2024-09-24

**Authors:** Jeetayu Biswas, Leora Boussi, Eytan Stein, Omar Abdel-Wahab

**Affiliations:** 1 Molecular Pharmacology Program, Sloan Kettering Institute, Memorial Sloan Kettering Cancer Center, New York, NY, USA; 2 Department of Medicine, Leukemia Service, Memorial Sloan Kettering Cancer Center, New York, NY, USA

## Abstract

Dysregulation of the flow of information from genomic DNA to RNA to protein occurs within all cancer types. In this review, we described the current state of understanding of how RNA processing is dysregulated in cancer with a focus on mutations in the RNA splicing factor machinery that are highly prevalent in hematologic malignancies. We discuss the downstream effects of these mutations highlighting both individual genes as well as common pathways that they perturb. We highlight examples of how alterations in RNA processing have been harnessed for therapeutic intent as well as to promote the selective toxicity of cancer cells.

## Teaser

This review describes the current state of understanding of how RNA processing is dysregulated in cancer (including alterations in RNA splicing, capping, polyadenylation, and decay) and how this knowledge is being harnessed for therapeutic intent.

## Introduction to RNA processing

The life of an RNA, from transcription to degradation, is filled with co- and posttranscriptional processing events. The highly regulated processes of transcription, splicing, capping, polyadenylation, and finally, translation and posttranslational processing have been studied for decades (reviewed in Hwang et al. [2024]; Biswas et al. [2021]; Vera et al. [2016]). More recently, the tools and resources have been developed to understand how these processes are altered in malignancy (reviewed in Bradley and Anczuków [2023]; Wheat and Steidl [2021]; Stanley and Abdel-Wahab [2022]; Dvinge et al. [2016]).

Initial expressed sequence tag libraries found an elevated frequency of stop codons in cancer-specific alternatively spliced transcripts (Chen et al., 2011). Further work has shown that tumors harbor 30% more alternative splicing events than normal tissues (Kahles et al., 2018). With the expansion of genomic and transcriptomic sequencing, many patients, particularly those with hematologic malignancies, have been found to have mutations in the core RNA processing machinery as well as alterations in downstream RNA processing (reviewed in Chen et al. [2021]; Stanley and Abdel-Wahab [2022]; Bradley and Anczuków [2023]). These alterations lead to effects on gene expression and resulting protein translation with a myriad of cellular consequences. Finally, systematic studies sequencing matched cancer and normal tissues (such as Genotype-Tissue Expression [GTEx] [GTEx Consortium, 2020; Battle et al., 2017] and The Cancer Genome Atlas [TCGA] [Kahles et al., 2018]) have found that tumor cells have an average of 30% more alternative splicing events than normal counterpart tissues (Kahles et al., 2018). Additionally, single nucleotide variants present within tumor cells have the potential to abolish or create splice sites (Supek et al., 2014; Jung et al., 2015) that impact splicing of an mRNA encoded from that gene in cis.

Here, we review some of the most well-studied examples of RNA processing alterations in cancer, focusing on the role of splicing factor mutations and splicing alterations in hematologic malignancies and other cancer types. We conclude with prior and ongoing attempts to therapeutically target these pathways and where future opportunities may be present.’

### RNA splicing

**Figure 1.**
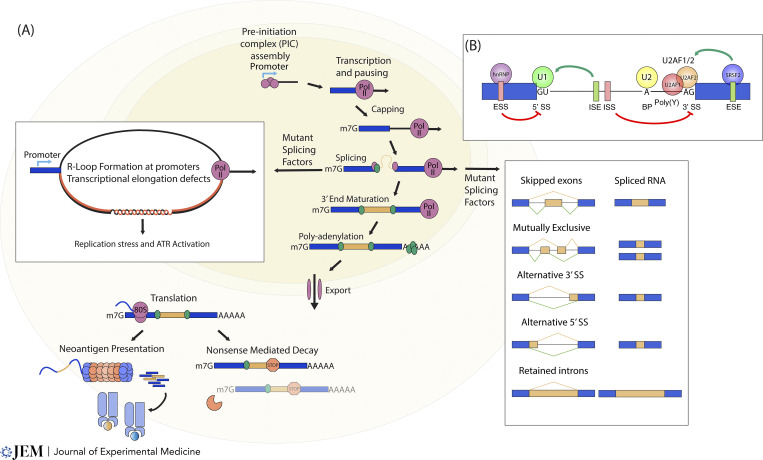
**Normal RNA splicing, 5′-end capping, and 3′-end cleavage and polyadenylation. (A)** Transcription and RNA processing begin with assembly of the pre-initiation complex on gene promoters, followed by recruitment of RNA polymerase II, and 5′ pausing. RNA transcription (blue bar), 5′ capping, and splicing occur cotranscriptionally on nascent RNA. These processes are facilitated by the recruitment of protein cofactors that facilitate capping, exon junction complex formation, and splicing (green dots). 3′-end maturation and polyadenylation then occur and release the RNA, allowing it to be exported into the cytoplasm. Splicing dysregulation, cellular stress, and the presence of mutant splicing factors can lead to the formation of tripartite DNA/RNA structures (R-loops) and downstream genomic instability. Different patterns of RNA splicing can occur (left: gold and green lines) and consequently lead to changes in the downstream RNA (right column). These changes can include the inclusion of novel sequences (gold bars) within the transcript of interest (blue bars). Once the mature alternatively spliced RNA is exported from the nucleus, it undergoes translation in the cytoplasm. Consequences of translation can lead to (left) translation of protein and eventual possible presentation on cell surface MHC molecules (blue and gold circles). Alternatively, the presence of a premature stop codon (red stop sign) can cause recruitment of the RNA degradation machinery (orange pac-man) and RNA downregulation through nonsense-mediated decay. **(B)** Binding of splicing factors to the RNA and regulation through cis and trans elements. Enhancers (green box, green arrow) and silencers (red box, red arrow) of splicing can be present in the intron (ISE, ISS) or exon (ESE, ESS). These sequences can be recognized by RNA binding proteins such as hnRNP (purple circle) or SRSF2 (blue circle) and promote (green arrow) or repress (red arrow) alternative splicing patterns.

Cotranscriptional regulation of RNA processing begins with 5′ capping of mRNA and recruitment of the spliceosome to define exons from introns (Fig. 1 A). The spliceosome is recruited to RNA as nascent RNA is transcribed in the nucleus. Splicing is regulated by a combination of RNA polymerase kinetics (Roberts et al., 1998; Eperon et al., 1988; Braberg et al., 2013) as well as characteristic sequence nucleotide motifs embedded in the RNA (Rogers and Wall, 1980; Shapiro and Senapathy, 1987; Senapathy et al., 1990) to perform two ligation reactions. The core sequence motifs in RNA demarcate the borders of the intron (known as 5′ and -3′ splice sites), the branchpoint nucleotide (which is most commonly an adenosine), and the polypyrimidine tract.

The spliceosome consists of RNA binding proteins (RBPs) and small nuclear RNAs that together form a small nuclear ribonucleoprotein complex (snRNP) (reviewed in Matera and Wang [2014]; Wilkinson et al. [2020]; Will and Lührmann [2011]). Over 95% of human genes undergo splicing, and most of these events are catalyzed by the major spliceosome (also known as the U2 spliceosome). A small fraction of the remainder of splicing events (<1% of human introns) are orchestrated by the minor spliceosome (also referred to as the U12 spliceosome) (reviewed in Patel and Steitz [2003]; Turunen et al. [2013]). These complexes carry out splicing through the recruitment of different snRNPs to different sequence motifs on an RNA molecule.

For the major spliceosome, early spliceosome assembly initiates as the U1 snRNP initially binds the 5′ splice site (most commonly a GT dinucleotide), SF1 binds the branchpoint sequence, U2AF1 binds the 3′ splice site (generally an AG dinucleotide), and U2AF2 binds to the adjacent polypyrimidine tract. Displacement of SF1 by the U2 snRNP allows for the SF3B1 component of U2 snRNP to recognize the branchpoint nucleotide (reviewed in Wilkinson et al. [2020]; Will and Lührmann [2011]; Matera and Wang [2014]).

The minor spliceosome is comprised of U11, U12, U4 atac, U6 atac, and ZRSR2, the latter of which is an X-chromosome encoded protein involved in 3′ splice site recognition for ∼1% of introns in the genome (reviewed in Patel and Steitz [2003]; Turunen et al. [2013]).

Following initial spliceosome assembly, the catalytic core of the spliceosome occurs through recruitment of the U4/U5/U6 tri-snRNP followed by lariat formation and cleavage. Once intron/exon boundaries are defined, the intron is removed via covalent nucleophilic attack of the 5′ end of the intron to the branchpoint nucleotide. The final product of splicing includes an excised lariat structure where the 5′ end is covalently linked to the 3′ branchpoint. A second covalent linkage ligates the 5′ end and 3′ end of RNA together and releases the lariat from the RNA where it is linearized by the debranching enzyme DBR1 and degraded.

In addition to core sequence motifs in RNA, additional layers of splicing regulation can be added by modulating RNA polymerase kinetics (as reviewed in Naftelberg et al. [2015]; Saldi et al. [2016]), recruitment of trans-acting RBPs, and utilization of cis-acting RNA sequences outside of the core splicing elements. Auxiliary sequences include splicing enhancers or silencers referred to as exonic or intronic splicing enhancers (ESE and ISE, respectively) or silencers (ESS or ISS) (Fig. 1 B). These sequences are bound by cognate RBPs with the heterogeneous nuclear ribonucleoprotein (hnRNP) family generally functioning as splicing silencers and serine/arginine-rich (SR) proteins functioning as splicing enhancers.

Processing of the RNA also includes the addition of the 5′ methyl-7-guanosine cap that protects the RNA from 5′ to 3′ exonucleases, assists with nuclear export of RNA, and functions to recruit translation initiation factors (Fig. 1 A). Cotranscriptional capping is performed by three enzymes—an RNA triphosphatase, RNA guanylyltransferase, and guanine-N7 methyltransferase (reviewed in Ramanathan et al. [2016]; Richter and Sonenberg [2005]).

Finally, once an RNA completes transcription, the transcript undergoes endonucleolytic cleavage followed by the addition of a 3′ poly(A) tail (Fig. 1 A). Alternative polyadenylation (APA) sites often have the canonical “AAUAA” polyadenylation sequence (PAS) but can utilize variants of this sequence with differing efficiencies. APA occurs in ∼70% of human genes and can lead to different 3′ termini from the same gene of interest. Like alternative splicing, APA exhibits tissue-specific regulation and is regulated by both cis-acting sequence elements and trans-acting RBPs. Most PAS sequences are in the 3′ UTR; however, intronic PAS sites occur and the process of intronic polyadenylation (IPA) can lead to truncated and nonfunctional RNAs (reviewed in Mitschka and Mayr [2022]).

Changes in alternative splicing can dramatically affect downstream gene expression through reading frame shifts or encoding alternative amino acids. Shifts in the reading frame will most often lead to the presence of a premature termination codon (PTC) and target the transcript for nonsense-mediated decay (NMD) (reviewed in Lareau et al. [2007]).

## Cancer-associated mutations in RNA processing machinery

High-throughput sequencing across cancer samples with matched normal controls at a large scale has been instrumental in discovering novel cancer driver mutations. Systematic analysis of the TCGA found putative driver mutations in 119 splicing factor genes (Seiler et al., 2018a). Mutations in the RNA processing machinery are highly prevalent in myeloid malignancies including acute myeloid leukemia (AML), chronic myelomonocytic leukemia (CMML), and myelodysplastic syndromes (MDS) (reviewed in Chen et al. [2021]; Stanley and Abdel-Wahab [2022]; Obeng et al. [2019]). Studies also found similarly high levels of mutational enrichment in chronic lymphocytic leukemia (CLL) as well as uveal melanoma and several other solid tumors (reviewed in Bradley and Anczuków [2023]).

**Figure 2.**
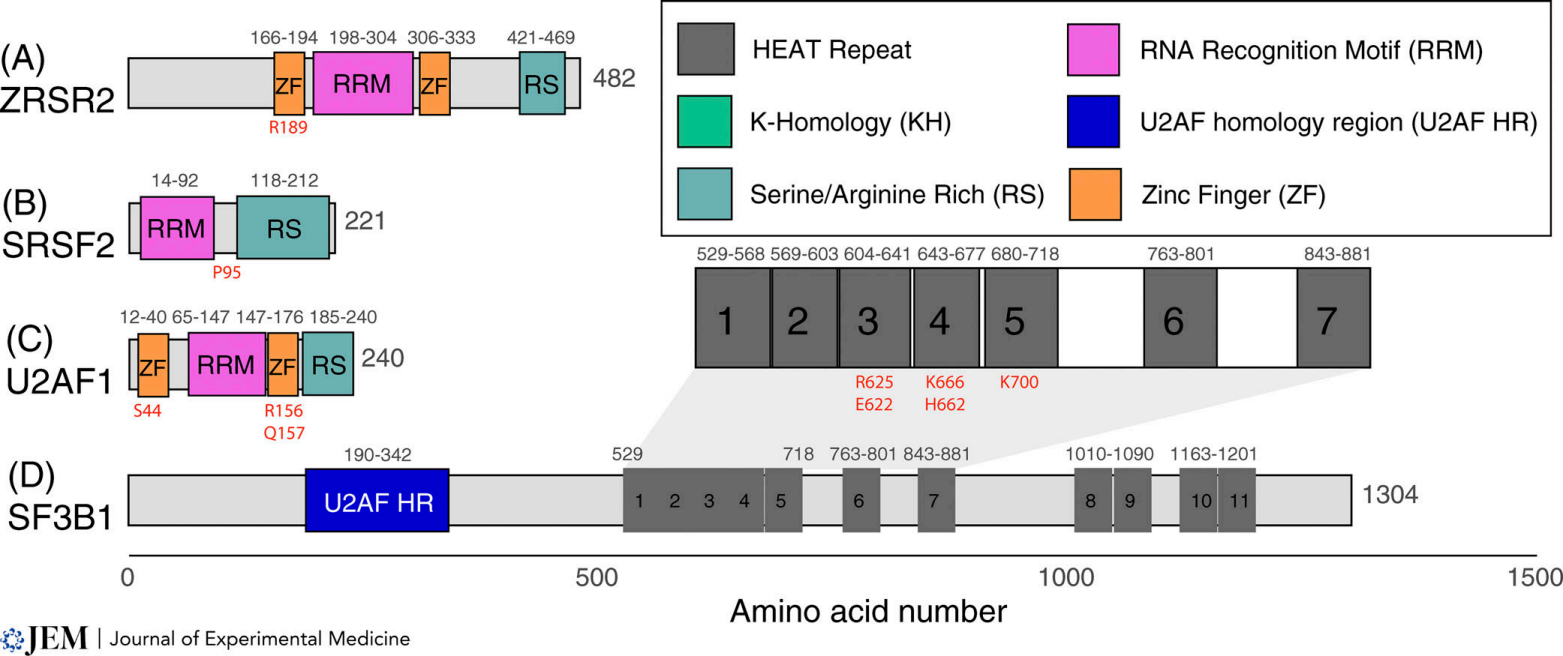
**RNA splicing factors frequently altered by somatic cancer-associated mutations. (A)** ZRSR2 contains multiple zinc finger domains (orange boxes) as well as an RRM domain (pink box) that are responsible for its interactions with RNA. The C-terminal RS domain facilitates its interactions with other splicing factors. Mutations in ZRSR2 have been described throughout the coding sequence; however, R189 (red letters) is a commonly affected residue. **(B)** SRSF2 contains an RNA recognition motif responsible for interactions with RNA substrates and an RS domain that facilitates its interactions with other splicing factors. Mutations in SRSF2 are located between these two domains at proline 95 (red letters). **(C)** U2AF1 contains multiple zinc finger domains (orange boxes) as well as an RRM domain (pink box) responsible for its interactions with RNA. The C-terminal RS domain facilitates its interactions with other splicing factors. Mutations in U2AF1 occur in each of its zinc finger domains (red letters). **(D)** SF3B1 contains an N terminal U2AF1 homology region (blue box) as well as multiple HEAT repeats (grey boxes) that facilitate binding to the branchpoint RNA as well as other protein–protein interactions. Zoomed in region containing common mutations in SF3B1 occur within HEAT repeats 3–5 (red letters). Legend (top right) highlights some of the common motifs that are found within the RNA-binding proteins that are discussed in this review.

In hematologic malignancies, splicing factors are particularly notable given the overall low tumor mutational burden in these diseases (Chalmers et al., 2017). Most frequent are mutations in the 3′-end processing machinery, namely mutations in SF3B1 and U2AF1, as well as the splicing enhancer SRSF2 and the minor spliceosome component ZRSR2 (Fig. 2, A–D). Apart from ZRSR2, where mutations confer loss of function, mutations affecting other commonly mutated splicing factors occur as heterozygous gain-of-function mutations (Yoshida et al., 2011; Madan et al., 2015). Also unique to ZRSR2 is its presence on the X chromosome, and accordingly, there is a male predominance in MDS patients bearing ZRSR2 mutations (Damm et al., 2012; Yoshida et al., 2011).

### SF3B1 mutations

The most frequently mutated splicing factor in cancer, SF3B1, is mutated across both solid and hematologic malignancies (Chen et al., 2021; Bradley and Anczuków, 2023; Kahles et al., 2018) (Fig. 2 D). SF3B1’s mutational frequency is particularly striking in MDS patients and in a particular subset where bone marrow evaluation identifies iron-laden mitochondrial deposits (ringed sideroblasts). This disease is historically called refractory anemia with ringed sideroblasts (MDS-RS) and over 75% of patients with MDS-RS have SF3B1 mutations (Papaemmanuil E. et al., 2011). The most recent classification of myeloid malignancies further emphasizes the importance of genetic driver mutations by classifying subsets by MDS by their driver mutation. In the most updated classifications, SF3B1 mutant MDS has been listed as its own disease subtype (Malcovati et al., 2020; Khoury et al., 2022). Additionally, adverse risk CLL also has frequent SF3B1 mutations (Wang et al., 2011). In solid tumors, 15–20% of uveal melanomas (Harbour et al., 2013; Martin et al., 2013), 5.6% of breast cancers (Ellis et al., 2012), and 4% of pancreatic ductal carcinomas harbor mutations in SF3B1 (Biankin et al., 2012).

The enrichment of SF3B1 mutations in MDS-RS has led to several groups finding that SF3B1 mutations lead to coordinated mis-splicing of the mitochondrial transporters TMEM14C and ABCB7, which results in iron sequestration within the mitochondria (Dolatshad et al., 2015, 2016; Clough et al., 2022; Alsafadi et al., 2016; Ochi et al., 2022). Analysis of SF3B1 mutant samples across TCGA, MDS patient samples, and CLL samples found commonly dysregulated RNAs (Inoue et al., 2019). The convergence of SF3B1 mutations on BRD9 mis-splicing and poison exon inclusion in BRD9 was found to disrupt non-canonical BAF chromatin remodeling and drive malignancy (Inoue et al., 2019).

SF3B1 and its U2 snRNP-associated cofactors determine the correct branchpoint nucleotides to use in splicing. Accordingly, mutations in SF3B1 or drugs that interact with SF3B1 cause aberrant branchpoint recognition. The most common cancer-associated mutations in SF3B1 (K700E, R625*, K666*) are located in the protein’s C-terminal HEAT repeats, and recent work has shown that these amino acids cluster into a pocket where the cofactor SUGP1 sits (Zhang et al., 2023). Loss of SUGP1, like the mutation of SF3B1, causes aberrant branch point selection and cryptic 3′-end splicing (Benbarche et al., 2024; Zhang et al., 2019). The aberrant 3′ splice sites promoted by mutations in SF3B1 are most often 10–30 nucleotides upstream of the canonical 3′ splice site. Mutant SF3B1 requires a different G-patch containing protein to enforce aberrant RNA splicing known as GPATCH8. When GPATCH8 is silenced, cryptic splice sites are reverted in the setting of the SF3B1 mutation (Benbarche et al., 2024).

While splicing changes are highly context dependent and vary across cell lines and cancer types, work intersecting human and mouse model systems containing SF3B1 mutations have found two SF3B1 mis-spliced target mRNAs that are highly conserved: MAP3K7 and PPP2R5A (Liu et al., 2020, 2021). Aberrant 3′ splice site usage in both targets caused downregulation of the downstream targets by NMD. MAP3K7 dysregulation affects downstream p65 and p38 MAPK signaling, and this, in turn, affects NF-κB (Liu et al., 2021) and GATA1 (Lieu et al., 2022) and leads to defects in erythroid differentiation. PPP2R5A encodes the alpha isoform of the B56 regulatory subunit of the serine/threonine phosphatase PP2A. SF3B1 mutations cause the degradation of PPP2R5A via NMD and lead to the upregulation of phosphorylated residues in a wide range of PP2A substrates including p65, AKT, MYC, and BCL2 (Liu et al., 2020). In addition to the aforementioned targets, several other targets of mutant SF3B1 have been described recently (Pellagatti and Boultwood, 2021; Zhou et al., 2020).

### SRSF2 mutations

SRSF2 is mutated in 50% of patients with CMML, 10–14% of patients with AML, and 20–30% of patients with MDS (Yoshida et al., 2011), where these mutations confer an increased risk of transformation from MDS to AML (Papaemmanuil et al., 2013).

Mutations in SRSF2 are centered at its proline 95 residue and alter RNA binding specificity favoring CCNG binding over GGNG nucleotides (Fig. 2 B). This change in RNA binding specificity promotes the inclusion of a C-rich poison exon in EZH2 that causes EZH2 mRNA transcript to undergo NMD (Kim et al., 2015). Consistent with this functional intersection, SRSF2 and EZH2 mutations are mutually exclusive in MDS patients (Kim et al., 2015). EZH2 encodes a histone methyltransferase, which is crucial for silencing of stem cell renewal genes, and this splicing-mediated downregulation of EZH2 alters hematopoiesis in a manner that promotes MDS (Sashida et al., 2014, 2016). Other aberrantly spliced targets of SRSF2 include transcription factors such as IKAROS and BCOR (the latter of which is frequently mutated in hematologic malignancies), the apoptosis regulator CASP8, and the tyrosine kinase FYN (Kim et al., 2015; Zhang et al., 2015).

### U2AF1 mutations

U2AF1 mutations occur mostly in myeloid malignancies where they are associated with high-risk MDS (Graubert et al., 2011; Haferlach et al., 2014; Papaemmanuil et al., 2013) and adverse-risk AML. U2AF1 mutations are also present in solid tumors (in particular non-small cell lung cancer [Imielinski et al., 2012]) and are most often located within one of its two zinc finger domains (S34 and Q157 [Yoshida et al., 2011; Graubert et al., 2011]) and affect cassette exon usage via their effects on the 3′ splice site recognition (Ilagan et al., 2015) (Fig. 2 C). Prior work has found that altered splicing by mutant U2AF1 can affect DNA damage response (through alterations in ATR and FANCA), epigenetic regulation (through alterations in H2AFY, ASXL1, BCOR, DNMT3B), apoptosis through CASP8 splicing, and innate immune signaling (through altered IRAK4 and Myd88) (Ilagan et al., 2015; Smith et al., 2019). U2AF2 is the dimeric partner of U2AF1. U2AF2 mutations are less common than U2AF1 mutations and often occur within the first two RNA recognition motifs (G176 and L187) (Haferlach et al., 2014).

In addition to the canonical function in splicing, noncanonical functions of U2AF1 and U2AF2 include a possible role in translation where the U2AF1/2 heterodimer has been proposed to function as a regulator of cytoplasmic mRNA translation (Palangat and Larson, 2012; Akef et al., 2020).

### ZRSR2 mutations

ZRSR2 loss-of-function mutations result in minor intron retention (Inoue et al., 2021; Madan et al., 2015), which most commonly results in NMD of the transcript. CRISPR knockout screens of minor intron-containing genes in mouse and human hematopoietic cell lines found that loss of the minor intron-containing gene LZTR1 conferred cytokine independence in all tested lines. LZTR1 encodes an adaptor for a ubiquitin-ligase that degrades the RAS-GTP protein RIT1, and interestingly, mutations within the LZTR1 minor intron or activating mutations in RIT1 have been described in the Rasopathy Noonan syndrome as well as myeloid leukemias (Inoue et al., 2021).

## Splicing and RAS signaling: KRAS splice variants and RAS-RAF splicing in cancer

Analyses integrating RNA splicing and binding profiles of mutant RNA splicing factors have attempted to find common pathways dysregulated by the gain-of-function mutations present in splicing factor mutant malignancies. Wheeler et al. identified aberrant RNA splicing events in induced pluripotent stem cell models created from patients with U2AF1 S34F or SRSF2 P95H mutations and further narrowed down this list using the binding profiles of the mutant proteins. From this analysis, a hyperactive isoform of the stimulator G protein alpha subunit (Gas-L) was found to activate ERK/MAPK signaling (Wheeler et al., 2022).

Beyond mutations in genes encoding RNA splicing machinery, there are numerous examples of mutations affecting RNA splicing in cis within genes with very well-established roles in cancer and, in particular, within the RAS/RAF/MAPK pathway. Here, we highlight key examples of such phenomenon by describing annotated splice variants and unannotated splicing alterations in genes encoding RAS and RAF proteins. For example, the gene encoding KRAS results in two alternatively spliced isoforms (KRAS4A and KRAS4B) that vary in the composition of exon 4, which encodes the C terminal membrane targeting region (Kochen Rossi et al., 2023; Nuevo-Tapioles and Philips, 2022). These isoforms regulate the membrane association of KRAS with different surfaces within the cell. While KRAS4B has been highly studied as the dominant isoform, more evidence has shown that KRAS4A may play an important role in malignancy by directly binding hexokinase 1 and localizing to the mitochondrial outer membrane (Amendola et al., 2019).

Mutations in KRAS (G12C, G12D, Q61K, and A146T) as well as the BRAF V600E mutation all drive resistance to the EGFR inhibitor Osimertinib (reviewed in Leonetti et al. [2019]). Interestingly, KRAS Q61K mutations were recently shown to introduce a cryptic splice donor site leading to aberrant splicing of exon 3 and a premature stop codon. Recently discovered concurrent G60 silent mutations eliminate the splice donor site, rescuing protein function, and lead to persistent ERK1/2 activation. Additionally, the region around Q61 is enriched in ESE motifs, and antisense oligonucleotides (ASOs) potentially can also silence the splice donor site and allow full-length KRAS Q61K protein formation. In preclinical models, ASOs targeting the ESE induced PTC formation and decreased KRAS Q61K activity as well as similar mutations in NRAS and HRAS mutant cancers (Kobayashi et al., 2022b).

The RAF kinase BRAF acts downstream of RAS, and when dimerized and activated, promotes cell survival. Oncogenic mutations in BRAF activate MAPK signaling and BRAF inhibitors prevent dimerization and activation of BRAF. In patients that have progressive disease in the presence of a BRAF inhibitor, resistant cells exhibit alternative splicing of BRAF into a 61-kDa protein lacking the exons that encode the RAS-binding domain (Poulikakos et al., 2011). This leads to enhanced dimerization, representing an important mechanism by which cancers can subvert oncogene inhibition. Follow-up work found that exon skipping occurs through a mutation 51 nt upstream of the 3′ splice site of intron 8, and splicing modulators that bind to SF3B1 (discussed below) were able to counteract the formation of this drug-resistant isoform (Salton et al., 2015). Most recently, there are also reports that deletions in genomic DNA can give rise to the aberrant drug-resistant BRAFV600E isoform (Aya et al., 2024).

At the transcript level, several other well-characterized examples of altered splicing occur in malignant cells and have been reviewed elsewhere (reviewed in Rahman et al. [2020]; Bradley and Anczuków [2023]).

## Common pathways in splicing altered cancers: R-loops and transcriptional elongation

Mutations of the aforementioned splicing factors are mutually exclusive with one another, and the mechanism as to how these disparate pathways lead to a common disease outcome is still being evaluated. One unifying theory suggests that these mutations affect the rate of transcriptional pause release and therefore lead to the formation of aberrant tripartite nucleic acid structures in the nucleus known as R-loops (Chen et al., 2018) (Fig. 1 A). Initial observations found that transcription defects caused impaired assembly of late-stage spliceosome components. Both R-loop formation and defective assembly can lead to the observed transcriptional elongation defects that have also been seen with spliceosomal mutations (Boddu et al., 2024). Increased presence of R-loops can lead to replication stress and activation of downstream ATR signaling (Chen et al., 2018) which prevents cell cycle progression in hematopoietic progenitors.

## Nonsense-mediated RNA decay

In approximately one-third of alternatively spliced transcripts, splicing can change RNA stability through the recruitment of different RBPs or through the incorporation of frameshift mutation resulting in the formation of PTCs (Lewis et al., 2003). PTCs are recognized by the NMD machinery and lead to RNA degradation of the alternatively spliced transcript. In addition to regulating the production of other proteins, splicing factors such as SRSF2 can utilize alternative splicing to autoregulate their protein levels through alternative inclusion of PTC-containing poison exons (Sureau et al., 2001).

## Splicing and the immune system: Neoantigen formation and chimeric antigen receptor T cell (CAR T) resistance

Both frameshift as well as in-frame mutations from splicing changes can lead to novel, unannotated peptides that become presented on the cell surface (either directly or bound to MHC class I or II). These immunogenic peptide sequences occur during the translation of alternative splicing events and when splice junctions border transposable elements (Merlotti et al., 2023; Burbage et al., 2023). Given the increased levels of alternative splicing in cancer, tumor-specific neoantigens that are derived from unannotated transcripts have the potential to give rise to peptides presented on MHC I molecules (Smart et al., 2018).

Cancer immunoediting is the process by which tumors and the immune system undergo dynamic changes and these changes can allow for escape from immune therapies such as CAR T cell therapy as well as antibody therapies (Dunn et al., 2002; Mittal et al., 2014). The anti-CD20 antibody rituximab is an integral part of treatment for lymphoma. The MS3A1 gene encoding CD20 produces four variant isoforms. In follicular lymphoma relapses following anti-CD20 antibody treatments, downregulation of CD20 was associated with CD20 splicing changes and ASOs modulating these isoforms in vitro can increase anti-CD20 antibody-mediated cytotoxicity, making this a possible therapeutic intervention for relapses that occur after anti-CD20–based immunotherapy (Ang et al., 2023).

CAR T cell therapies have become a mainstay for treating lymphoid malignancies. CAR T cells utilize an engineered extracellular antigen recognition domain that recognizes tumor-specific antigens paired with an intracellular signal transduction domain to promote T cell activation, expansion, and killing of antigen-positive cells. The first FDA-approved CAR T cells were directed against the B cell antigen CD19. Post-anti-CD19 CAR T relapse mechanisms utilize a broad array of mechanisms that impede the presentation of the normal CD19 epitope to the cell surface. A significant portion of cancers occurring at relapse from CAR T cell therapy are target antigen-negative. Similar CD19 negative relapses are also seen with the CD19xCD3 bispecific antibody blinatumomab. While it was originally thought that the majority of CD19 negative relapses occur by frameshift mutations with the gene encoding CD19 (Orlando et al., 2018), others have suggested that these mutations are subclonal and do not result in total loss of antigen at the surface (Sotillo et al., 2015). Mechanisms of resistance include skipping of exon 2, which causes ER sequestration and lack of expression at the cell surface, and skipping of exons 5 and 6, which encode the transmembrane domain and lead to intracellular trafficking defects (Sotillo et al., 2015). Aberrant splicing of the B cell immunotherapy target CD22 occurs via exon 2 skipping and can lead to exclusion of the start codon and loss of exon 5 and 6, resulting in loss of the CAR T immunoepitope (Zheng et al., 2022). Further splicing-dependent mechanisms of resistance are likely to be discovered as CAR T therapies continue to increase in use.

## APA and IPA

Cleavage and polyadenylation (CPA) of RNA is accomplished through recruitment of the multiprotein CPA machinery to change the 3′ end of an RNA transcript (Fig. 1 A). CPA specificity factor (CPSF) recognizes polyadenylation sites via the canonical AAUAAA sequence motif embedded in pre-mRNA. Additional sequence elements including UGUA and downstream U-rich or G+U-rich sequences are recognized by cleavage factor I (CFI) and cleavage stimulation factor (CSTF).

APA causes alternative 3′ UTR formation. When cleavage and polyadenylation occur within an intron, also referred to as IPA, this causes NMD or truncation of the protein and can also lead to an alternative 3′ UTR. While APA can change transcript stability, both APA and IPA can lead to decreased protein translation. Some prominent examples of APA dysregulation in tumors include global 3′ UTR shortening (Mayr and Bartel, 2009; Sandberg et al., 2008; Xia et al., 2014). One factor, NUDT21/CPSF5, has been shown to be downregulated in malignancy and confer poor prognosis. Downregulation of NUDT21/CPSF5 has been shown to result in 3′ UTR shortening for nearly 1,500 genes (Sun et al., 2017; Chu et al., 2019; Xing et al., 2021).

Premature CPA can occur in malignancies through mutation of CPA factors, changes in transcription elongation (Dubbury et al., 2018; Krajewska et al., 2019), or alterations in splicing factors. Several reviews have covered transcript-specific alterations in cleavage and poly-adenylation that occur in malignancy (reviewed in Obeng et al. [2019]; Mitschka and Mayr [2022]).

## Decapping

After 5′-to-3′ decay of mRNA, Decapping Scavenger (DCPS) enzyme hydrolyzes m7Gpp to release m7Gp and the monophosphorylated RNA (Fig. 1 A). CRISPR screening in AML mouse models found the RNA decapping scavenger DCPS to be an important vulnerability both in vitro and in vivo (Yamauchi et al., 2018; Yoshimi and Abdel-Wahab, 2018). Small molecules targeting DCPS have been employed in early trials for spinal muscular atrophy (SMA) (Gogliotti et al., 2013), and more recent development of proteolysis targeting chimeras (PROTACs) targeting DCPS (Swartzel et al., 2022) may hold future promise as a therapeutic in AML.

## Targeting RNA processing in hematologic malignancies

**Figure 3.**
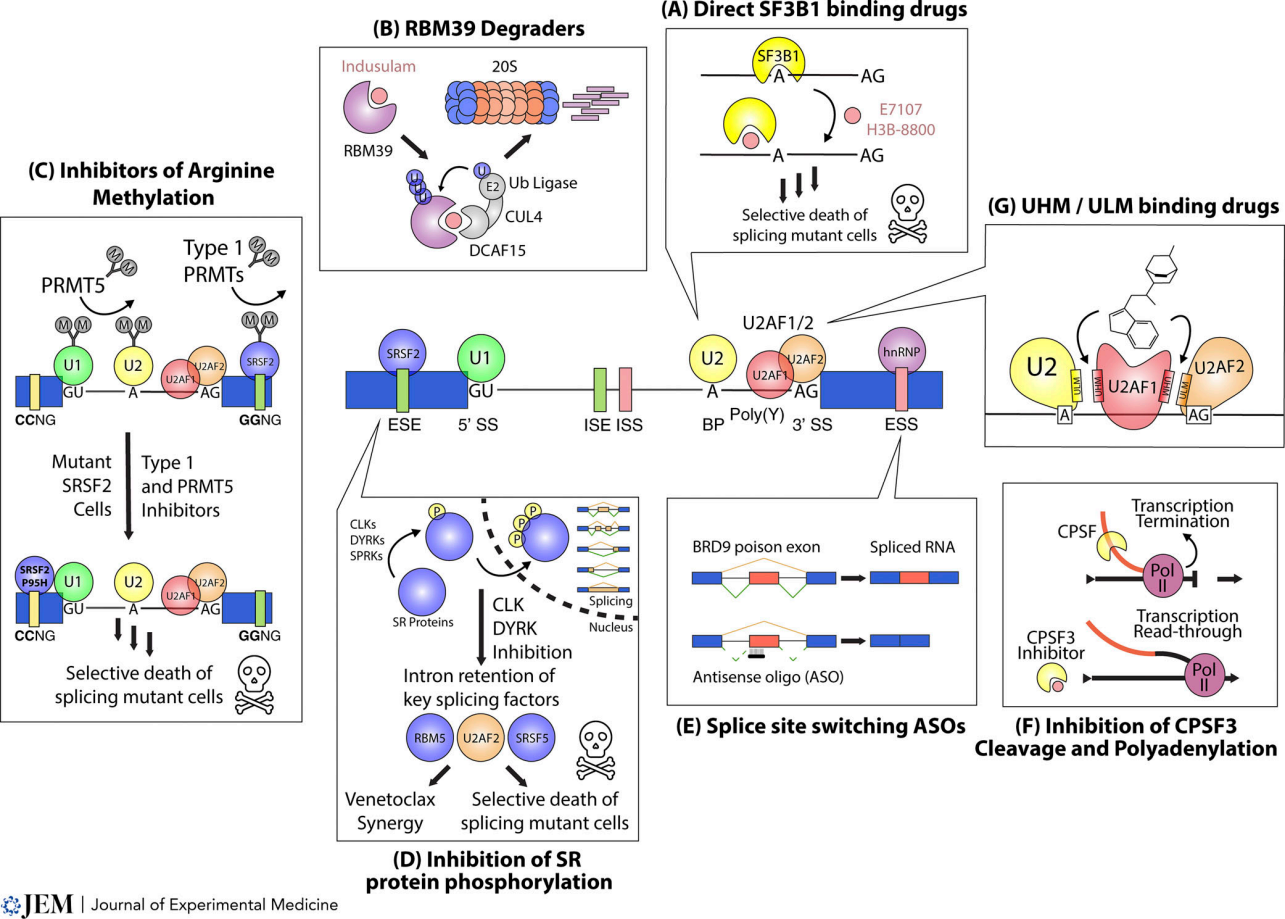
**Therapeutic approaches described to date for pharmacologic modulation of RNA splicing. (A)** The binding of small molecules (pink circle) to the HEAT motifs of SF3B1 (yellow pac-man) can prevent recognition of canonical branchpoint sequences and cause preferentially killing of splicing factor mutant cells in pre-clinical models. **(B)** Small molecule sulfonamides (pink circle) can recruit the ubiquitination (Ub) machinery (grey) to the splicing factor RBM39 and promote its degradation via the 20S proteasome (orange and blue barrel). **(C)** Arginine methylation (grey circles) is a posttranslational modification (colored circles) that occurs in numerous RNA splicing factors. Inhibition of either type 1 PRMT or PRMT5 enzymes leads to loss of arginine methyl marks (downward arrow) and consequent preferential cell death of splicing factor mutant cells in preclinical models. **(D)** Phosphorylation of SR proteins (blue circles) by CLK, DYRK, or SPRK enzymes can affect their translocation into the nucleus where they function to regulate splicing. Inhibition of CLK or DYRK enzymes leads to decreased phosphorylation and intron retention within key splicing factors (colored circles) and can synergize with BCL2 inhibition to lead to preferential cell death of splicing factor mutant cells. **(E)** Antisense oligonucleotides (black bar) can be used to block splice sites (dashed lines) to favor certain splicing outcomes (solid lines) leading to preferential alternative splicing patterns (blue and red bars). **(F)** Small molecules (pink circles) targeting the polyadenylation and 3′-end processing machinery can prevent the normal transcription termination of an RNA (orange line). This inhibition can lead to transcriptional readthrough (orange and black line) and downstream RNA destabilization and downregulation. **(G)** Small molecule compounds (depicted in stick structure) can block (arrows) the interactions between key components of the core splicing machinery containing UHM domains (colored blobs).

Targeting of RNA processing has been pursued for a number of diseases over the last 15 years. While early success has been seen with ASOs in neurologic conditions such as SMA (Rigo et al., 2012; Hua et al., 2010), the field remains in its infancy, and we highlight a few of the early attempts to modulate RNA processing using a variety of therapeutic approaches that have entered clinical trials below (Fig. 3 and Table 1).

**Table 1.** Summary of therapies targeting RNA splicing described in Fig. 3

Target	Agent	Phase	Patient population	References
SF3B1	H3B-8800	1	MDS, AML, CMML	Steensma et al. (2021)
RBM39 degrader	Indisulam + idarubicin and cytarabine	2	R/R AML, HR-MDS	Assi et al. (2018)
	E7820	2	R/R splicing factor-mutant AML, MDS, or CMML	Bewersdorf et al. (2023b)
PRMT inhibitor	GSK3326595 monotherapy or + azacitidine	1/2	R/R MDS, CMML, AML evolved from prior MDS	Watts et al. (2019)
	PRT543	1	MDS, MF	Patel et al. (2021)
	JNJ-64619178	1	Low-risk MDS	Haque et al. (2021)
CLK inhibitor	CTX-712	1/2	R/R AML, HR-MDS	Al-Kali et al. (2023), Shimizu et al. (2022)
ASO	BP1001 monotherapy or + cytarabine	1	AML, MDS, CML-BP	Ohanian et al. (2020)
	Cenersen (EL625) + cytarabine and idarubicin	2	R/R AML	Alvarado et al. (2007)
	Cenersen (EL625) + idarubicin with or without cytarabine	2	R/R AML	Cortes et al. (2012)
RNA capping	Ribavirin + cytarabine	1	R/R AML	Assouline et al. (2015)
RNA polyadenylation	JTE-607 targeting of CPSF3	N/A	Preclinical	Ross et al. (2020)
UHM/ULM	NSC 194308	N/A	Preclinical	Chatrikhi et al. (2021)

Abbrevations: BP: blast phase. HR-MDS: high-risk MDS. MF: myelofibrosis. R/R: relapsed/refractory.

### Targeting capping

The 5′ end of the RNA is often capped within cells, the cap interacts with the translation factor eIF4E and interactions with the ribosome-associated translation factor eIF4G allow for effective recruitment of RNAs to the ribosome (reviewed in Richter and Sonenberg [2005]). Cancers overexpress eIF4E and this increases the fraction of capped transcripts. Additionally, overexpression of eIF4E by itself can lead to oncogenic transformation (Lazaris-Karatzas et al., 1990). Ribavirin, a guanosine analog, suppresses eIF4E-mediated oncogenic transformation by mimicking the RNA cap (Kentsis et al., 2004). A phase I trial found that combination therapy of ribavirin with cytarabine was well tolerated in relapsed/refractory AML (Assouline et al., 2015). Targeting the RNA decapping enzyme DCPS with the small molecule RG3039 has been pursued clinically now and a phase 1 clinical trial found that the compound was well tolerated (Van Meerbeke et al., 2012).

### Targeting RNA splicing factors directly

Several natural product-derived small molecules have been found to bind the SF3b complex and interact with the branch site binding pocket of SF3B1 (Fig. 3 A) as well as other SF3b components such as PHF5A (Kotake et al., 2007). Preclinical data of these compounds has shown synthetic lethality with splicing factor mutant leukemia cells in vitro and in vivo (Seiler et al., 2018b; Lee et al., 2016). The largest clinical data to date with an SF3b inhibitor was with the orally available SF3B1 targeting agent, H3B-8800. While this agent was well tolerated, unfortunately, clinical trials in >50 patients with transfusion-dependent SF3B1 mutant MDS had limited clinical activity (Steensma et al., 2021).

Unbiased CRISPR screening targeting RBPs identified the splicing factor RBM39 as essential for AML maintenance (Wang et al., 2019). RBM39 degraders, the anti-cancer sulfonamides indisulam (E7070), E7820, and chloroquinoxaline sulfonamide, selectively degrade RBM39 by promoting interaction of RBM39 and the adapter protein DCAF15 with the CUL4/Db1 E3 ubiquitin ligase (Han et al., 2017; Uehara et al., 2017; Du et al., 2019) (Fig. 3 B). Prior clinical trials showed excellent safety profiles and some antitumor efficacy in solid tumors (Owa et al., 1999; Supuran, 2003; Mita et al., 2011).

Phase 2 trials combining indisulam, idarubicin, and cytarabine in relapsed/refractory AML and high-risk MDS showed efficacy in a heavily pretreated population (Assi et al., 2018). However, a phase 2 trial of 12 patients evaluating E7820 in splicing factor mutant AML, MDS, or CMML did not meet its primary endpoint of >1 patient achieving an objective response (Bewersdorf et al., 2023a).

### Targeting splicing factor posttranslational modifications

Drug screening for selective lethality in SRSF2 mutant leukemic cell lines found that in addition to direct inhibition of SF3B1 (as described above), drugs inhibiting symmetric or asymmetric arginine dimethylation preferentially killed spliceosome mutant cells (Fong et al., 2019). These drugs target protein arginine methyltransferases (PRMTs), and activity was seen individually with PRMT5 targeting as well as type 1 PRMT targeting compounds that target PRMT 1, 3, 4, 6 (Fig. 3 C). While phase 1 clinical trials have evaluated PRMT inhibitors in advanced solid cancers and lymphoma (El-Khoueiry et al., 2023; Ferrarotto et al., 2024; McKean et al., 2021) and suggested acceptable toxicity (Rodon et al., 2024), further trials are pending for splicing factor mutant cancers (Monga et al., 2023; Patel et al., 2021).

CDC2-like kinases (CLKs), serine/arginine protein kinases (SPRKs), and dual-specificity tyrosine-regulated kinases (DYRKs) are all involved in the phosphorylation of SR-containing proteins including SRSF2 (Fig. 3 D). SM08502 (cirtuvivint) competes for the ATP binding site of CLK1-4 and DYRK1-4. This agent exhibits activity across a variety of splicing factor mutant cell lines and xenograft models as well as synergy with venetoclax (Wang et al., 2023). Phase 1 clinical trials have revealed that cirtuvivint is well tolerated with evidence of antitumor activity in prostate cancer (Tolcher et al., 2021; Scott et al., 2022). A related CLK/DRYK inhibitor CTX-712 showed single-agent activity in both ovarian cancer and MDS/AML. Intriguingly, in the phase 1 trial of CTX-712, two of five relapsed/refractory AML patients and one of one MDS patient achieved complete remission (Yokoyama et al., 2022). Numerous clinical trials of additional CLK/DYRK inhibitors, either alone or in combination with other therapeutics, are now entering phase 1 trials.

### Targeting splicing regulatory sequencing with ASOs

ASOs provide site-specific base pairing, can be utilized to mask cis-acting splicing regulatory elements, and therefore lead to downstream alterations in alternative splicing. While major success was found using ASOs targeting SMN2 transcript to treat SMA, several approaches were attempted prior to clinical trials that targeted an hnRNP A1/A2 ISS (Hua et al., 2008, 2010). ASO therapy is in the early stages of oncology due to issues around systemic ASO delivery and penetrance in hematologic and metastatic cancer sites. To target the JAK-STAT pathway, STAT3-targeting ASOs have been tested in clinical trials for lung cancer and lymphoma (Reilley et al., 2018) and were well tolerated and showed disease activity (Roschewski et al., 2023; Hong et al., 2015). MDM4 targeting ASOs have been shown to lead to exon 6 skipping, decreasing MDM4 levels, and therefore increasing p53 tumor suppressor function (Dewaele et al., 2016). Additionally, MDM4 may work downstream of PRMT5 as a sensor of splicing defects to increase p53 response (Bezzi et al., 2013). Additionally, ASOs targeting aberrant BRD9 splicing (Fig. 3 E) in uveal melanoma (Inoue et al., 2019) have been shown to restore BRD9 protein levels preclinically. Further work is needed to determine how ASOs fare in clinical trials. Some early attempts to use ASOs targeting BCL2 in combination with chemotherapy (Walker et al., 2021) have shown that they are safe but did not improve outcomes compared with chemotherapy alone.

## Conclusions and future therapeutic opportunities

Several promising opportunities exist to target RNA processing. While some of the aforementioned approaches will continue into phase 2 clinical trials in the coming years, other early-stage approaches targeting splicing include small molecules targeting the U1 snRNP (Prajapati et al., 2023), polyadenylation (Fig. 3 F) (Araki et al., 2018), U2AF homology motifs (UHMs), and U2AF ligand motifs (Fig. 3 G) (ULMs) (Jagtap et al., 2020; Kobayashi et al., 2022a; Chatrikhi et al., 2021). Preclinical efficacy for molecules targeting the cleavage and polyadenylation complex component CPSF3 (Tao et al., 2024; Liu et al., 2023) has shown efficacy in leukemia (Ross et al., 2020), Ewing’s sarcoma (Ross et al., 2020), ovarian (Shen et al., 2023), and pancreatic cancer models (Alahmari et al., 2024). Additionally, recently developed synthetic introns that deliver a therapeutic payload that is spliced and expressed only in splicing factor mutant cells (North et al., 2022) are under development. Finally, additional preclinical studies are underway to determine if modulation of splicing can induce the generation of mis-splicing–derived immunogenic peptides and lead to increased response to checkpoint inhibitors (Lu et al., 2021).

While prior approaches have utilized synthetic lethality to target preferential cell death in spliceosome mutant cells (Seiler et al., 2018b), these have been limited by toxicity (Eskens et al., 2013; Reilley et al., 2018) and uncertain therapeutic windows in vivo (Steensma et al., 2021). Rather than targeting core spliceosomal components, approaches that utilize the context of spliceosome mutants, either by targeting mutant-specific cofactors (Benbarche et al., 2024) or by selectively killing mutant cells without affecting wild-type cells (North et al., 2022), may overcome some of the previous issues.

We are undoubtedly in an exciting time as our understanding of RNA processing in cancer biology advanced rapidly by increased utilization of high throughput sequencing, genome editing, and investments to target these pathways in early-stage clinical trials. In the coming years there will likely be an expansion of tools and small molecules to modulate RNA processing. Further mechanistic work is needed to connect the plethora of splicing changes that occur in cancer cells to the progression of malignancy and to develop a better understanding of how other aspects of RNA processing (such as capping) may be altered in cancer and targeted. By further understanding the downstream effects of this modulation, be it alternative splicing, polyadenylation, capping, NMD, or neopeptide presentation, we are getting closer to utilizing these tools to selectively eliminate cancer cells.
